# Opposite Effects of Single-Dose and Multidose Administration of the Ethanol Extract of Danshen on CYP3A in Healthy Volunteers

**DOI:** 10.1155/2013/730734

**Published:** 2013-10-08

**Authors:** Furong Qiu, Jian Jiang, Yueming Ma, Guangji Wang, Chenglu Gao, Xinfeng Zhang, Liang Zhang, Songcan Liu, Min He, Leilei Zhu, Yujie Ye, Qiuye Li, Ping Miao

**Affiliations:** ^1^Lab of Clinical Pharmacokinetics, Shuguang Hospital Affiliated to Shanghai University of Traditional Chinese Medicine, Shanghai 201203, China; ^2^Lab of Pharmacokinetics, Shanghai University of Traditional Chinese Medicine, Shanghai 201203, China; ^3^Key Lab of Drug Metabolism & Pharmacokinetics, China Pharmaceutics University, Nanjing 210038, China

## Abstract

The aim of this study was to investigate the effect of single- and multidose administration of the ethanol extract of danshen on in vivo CYP3A activity in healthy volunteers. A sequential, open-label, and three-period pharmacokinetic interaction study design was used based on 12 healthy male individuals. The plasma concentrations of midazolam and its metabolite 1-hydroxymidazolam were measured. Treatment with single dose of the extract caused the mean *C*_max_ of midazolam to increase by 87% compared with control. After 10 days of the danshen extract intake, the mean AUC_0–12_, *C*_max_, and *t*_1/2_ of midazolam were decreased by 79.9%, 66.6%, and 43.8%, respectively. The mean clearance of midazolam was increased by 501.6% compared with control. The in vitro study showed that dihydrotanshinone I in the extract could inhibit CYP3A, while tanshinone IIA and cryptotanshinone could induce CYP3A. In conclusion, a single-dose administration of the danshen extract can inhibit intestinal CYP3A, but multidose administration can induce intestinal and hepatic CYP3A.

## 1. Introduction

Danshen, the dried root of *Salvia miltiorrhiza*, has been used for hundreds of years to treat coronary heart and cerebrovascular disease [1]. The herbal medicine is also available as a prescription or an over-the-counter drug in countries such as China, Singapore, Korea, Janpan, Russia, Cuba, and South Africa and as a dietary supplement in the United States [2–6]. Danshen as a medicine or dietary supplement is often administered in combination with therapeutic drugs, causing clinically important herb-drug interactions and adverse outcomes [3]. Thus, safety of administrated danshen is of great concern. At present, many danshen preparations (e.g., crude drug, lipophilic extract, and hydrophilic extract) are commercially available, and the ethanol extract of danshen rich in lipophilic constituents is commonly used in Chinese clinics. The main constituents in the extract are tanshinones including cryptotanshinone, tanshinone IIA, tanshinone I, and dihydrotanshinone I.

CYP enzymes (CYPs) play an important role in detoxification and systemic clearance of xenobiotics. Of >55 human CYP isozymes presently known, CYP3A is considered to be the most important drug-metabolizing enzyme. It participates in metabolism of >60% of all marketed drugs. As the critical role of CYP3A in drug metabolism, inhibition or induction of this enzyme often leads to drug interactions [5]. In recent years, some studies revealed the effect of danshen extract on CYP3A4, especially the danshen extract rich in lipophilic constituents. Kuo et al. reported that the ethyl acetate extract of danshen could induce CYP3A in C57BL/6J mice [7]. By using a reporter gene assay and RT-PCR, Yu et al. demonstrated that cryptotanshinone and tanshinone IIA in the ethanol extract of danshen could activate the human pregnane and xenobiotic receptor (PXR) and consequently induce the expression of the *CYP3A4* gene [8]. It was found in the in vitro study using human liver microsomes that the ethanol extract of danshen had a significant inhibition toward CYP3A4-mediated midazolam metabolism [9]. 

The aim of this study was to investigate the effect of single- and multi-dose administration of the ethanol extract of danshen on the in vivo CYP3A activity in healthy volunteers. The constituent(s) inhibited or induced to CYP3A was also investigated using human liver microsomes and human cryopreserved hepatocytes. It will provide valuable information for using the danshen preparation in clinical practice.

## 2. Meterials and Methods 

### 2.1. Meterials

**Figure 1:**
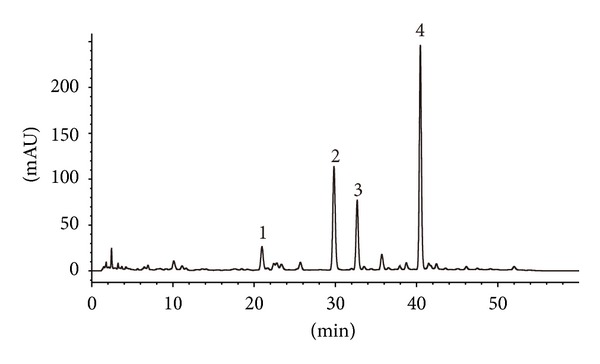
Representative chromatograms fingerprint of the danshen ethanol extract: (1) dihytdoxytanshinone I; (2) crytanshinone I; (3) tanshinone I; (4) tanshinone IIA.

Tanshinone I, cryptotanshinone, tanshinone IIA, and dihydrotanshinone I were purchased from the National Institute for the Control of Pharmaceutical and Biological Products (Beijing, China). NADPH, midazolam, and 1-hydroxymidazolam were purchased from Sigma-Aldrich (St. Louis, MO). HPLC-grade acetonitrile, methanol, and ethyl acetate were obtained from Merck (Darmstadt, Germany). Deionized water was purified using a Milli-Q system (Millipore Corporation, Billerica, MA). Midazolam tablets (15 mg/tablet, Lot 20100801) were manufactured by Jiangsu Nhwa Pharma Corporation. The ethanol extract of danshen in the form of capsule (250 mg/capsule, Lot 20090904) was manufactured, and the quality control was established and enforced strictly by Hebei Xinlong XiLi Pharmaceuticals Ltd according to state drug standard (China State Food and Drug Administration, Ws3-B-3140-98-009). The lipophilic components (tanshinone I, tanshinone IIA, cryptotanshinone, and dihydroxytanshinone I) and fingerprint of the ethanol extract of danshen were determined by HPLC on a C18 column and with a mobile phase of 20 mmol ammonium acetate : acetonitrile (30 : 70, V/V). The detection wavelength was set to 270 nm. For determination of fingerprint of the ethanol extract of danshen, a gradient mobile phase was used. The fingerprint was showed in Figure 1, and the contents of tanshinone IIA, cryptotanshinone, tanshinone I, and dihydrotanshinone I were 106.2 mg/g, 88.0 mg/g, 53.1 mg/g, and 13.5 mg/g, respectively. 

Human liver microsomes (10-donor pool, mixed gender) and human cryopreserved hepatocytes (Lot ONO and JYM, male donors) used in this study were provided by the Research Institute for Liver Disease Co. (Shanghai, China). 

### 2.2. Subjects

The clinical protocol and informed consent form were approved by the independent ethics committee of Shuguang Hospital affiliated to Shanghai University of Traditional Chinese Medicine. 12 healthy male volunteers were enrolled in the study after obtaining written informed consent.

All subjects were nonsmokers and judged to be healthy according to their medical histories, complete physical examinations, electrocardiograms, and routine laboratory test results. Subjects abstained from consuming herbal and citrus fruit products for 2 weeks before the study and from alcohol and medications for 2 weeks before and during the study period; caffeine-containing foods and beverages were also excluded during the study period. 

### 2.3. Pharmacokinetics of Midazolam in Healthy Volunteers after Oral Administering of Midazolam

The study design was a sequential, open-label, and three-period trial [10] conducted at the Shuguang Hospital phase I clinical trial ward. On the morning of day 1, the volunteers took a single dose of 15 mg of midazolam. Beginning on day 2, they received the ethanol extract of danshen (1 g, three times a day) for 10 days. On day 12, the volunteers received 1 g danshen extract 0.5 h before taking 15 mg midazolam. After a 3-week washout period, a single dose of the danshen extract (1 g) was administered 0.5 h before taking 15 mg of midazolam. Midazolam pharmacokinetic study days followed an identical course: the volunteers were fasted overnight before each dosing. The subjects were provided a light standard meal 4 h after medication intake and at 6 PM on the three test days of taking midazolam. Smoking and consumption of alcohol, coffee, tea, and any drugs were prohibited during the study. Four milliliters of blood was sampled from the forearm veins at 0, 0.25, 0.5, 0.75, 1, 1.5, 2, 2.5, 3, 4, 6, 8, and 12 h after taking midazolam and kept in heparinized Eppendorf tubes. The blood samples were centrifuged, and plasma was separated and stored at −80°C until the time of analysis. 

### 2.4. Analysis of Midazolam and 1-Hydroxymidazolam in Plasma Samples

Plasma samples were spiked with an internal standard (diazepam) and extracted with ethyl acetate. After evaporation of the organic solvent under nitrogen, reconstituted residues of the organic phase were analyzed using a liquid chromatography-tandem mass spectrometry (LC-MS/MS) system (API 4000, Applied Biosystems/SCIEX, CA). Chromatographic separation of the compounds was accomplished using a C_18_ column (5 *μ*m, 4.6 mm × 150 mm, Agilent) with water phase (ammonium acetate 4 mmol/L and methanoic acid 0.08%) : methanol (10 : 90, v : v) as the mobile phase at a flow rate of 0.80 mL/min. The MS/MS system was operated in positive ion electrospray ionization. The multiple reaction monitoring (MRM) detection mode was applied to midazolam (*m*/*z*: 326.4→291.2), 1-hydroxylmidazolam (*m*/*z*: 341.8→324.0), and diazepam (*m*/*z*: 285.2→193.1). The collision energy (CE), declustering potential (DP), and collision cell exit potential (CXP) were set as follows: midazolam: 35.88 V, 105.14 V, and 13.00 V, respectively; 1-hydroxymidazolam: 33.00 V, 91.05 V, and 17.94 V, respectively; and diazepam: 43.00 V, 98.06 V, and 11.45 V, respectively. 

The quantitative range for measuring midazolam and 1-hydroxymidazolam was 0.1 ng/mL to 150 ng/mL. The accuracy, precision, recovery, and stability tests all met the requirements for quantitative determination in biological samples. No matrix effect existed in this LC-MS/MS method.

### 2.5. Analysis of Tanshinones in Plasma Samples

For tanshinones, tanshinone I, cryptotanshinone, tanshinone IIA, and dihydrotanshinone I levels were determined by LC-MS/MS as previously described [11–13]. The plasma extraction method, chromatographic column, mobile phase, and instruments were the same as those previously mentioned. The mass spectrometer was operated in the positive ion mode, and quantification was thus performed using the MRM of the transitions of *m*/*z* 277.1→249.0 for tanshinone I, 297.3→251.2 for cryptotanshinone, 295.2→249.2 for tanshinone IIA, 279.2 →233.2 dihydroxytanshinone I, and 285.2→193.1 for diazepam. CE, DP, and CXP were set as follows: tanshinone I: 29.00 V, 98.06 V, and 16.32 V, respectively; cryptotanshinone: 43.00 V, 123.00 V, and 15.12 V, respectively; tanshinone IIA: 27.36 V, 103.66 V, and 15.00 V, respectively; dihydrotanshinone I: 28.32 V, 112.07 V, and 6.92 V, respectively; and diazepam: 43.00 V, 98.06 V, and 11.45 V, respectively.

### 2.6. Inhibition of CYP3A Activities by Danshen Ethanol Extract and Dihydrotanshinone I in Human Liver Microsomes [14]

HLMs used in this study were provided by the Research Institute for Liver Disease Co. (Shanghai, China). The microsomes were prepared from ten individual human donor livers.

The CYP3A enzymatic activities were characterized based on reaction of midazolam 1-hydroxylation. Incubation mixtures were prepared in a total volume of 200 *μ*L as follows: HLMs (1 mg/mL) 40 *μ*L, 1 mM NADPH 10 *μ*L, 100 mM phosphate buffer (pH 7.4) 130 *μ*L, 70 *μ*M midazolam 10 *μ*L, and a range of concentrations of tested compound 1 *μ*L. There was a 5 min preincubation period at 37°C before the reaction was initiated by adding the NADPH. The reactions were conducted for 5 min. Triplicate samples were run to generate IC_50_ value by incubating midazolam at 3.5 *μ*M in the presence of five concentrations of ethanol extract of danshen (final concentrations 0.2–100.0 *μ*g/mL) and dihydrotanshinone I (final concentrations ranging from 0.5 to 100.0 *μ*M) in the incubation mixture. Ethanol extract of danshen and dihydrotanshinone I were dissolved in DMSO (final concentration 0.5% in HLMs).

### 2.7. Induction of CYP3A4 mRNA by Tanshinones in Human Hepatocytes

Human cryopreserved hepatocytes were thawed in hepatocyte thawing medium and were seeded in collagen I precoated 24-well plates, with each well having a cell density of 3.0 × 10^5^ viable cells in 0.25 mL of hepatocyte plating medium. Viability as determined by trypan blue exclusion was 85% or better for this study. The cells were maintained at 37°C in a humidified incubator with 90% atmospheric air and 5% CO_2_. Twenty-four hours after isolation, plating, and incubation, hepatocytes were treated with vehicle, which contained the same amount of DMSO (0.1%), tanshinones (2 *μ*M), and rifampicin (25 *μ*M) for 72 hours. All drugs were dissolved in DMSO and then added to the culture medium (final DMSO concentration, 0.1%). Incubation medium containing 0.1% DMSO served as the vehicle control. After daily treatment for 3 days, the medium was removed, and the cells were washed with saline. Total RNA was isolated from cells using TRIzol reagent (Invitrogen) according to the manufacturer-supplied protocol. Quantitative real-time PCR was performed using gene-specific primers and the SYBR Green PCR kit (Invitrogen) in an ABI 7900 system (Applied Biosystems). The relative quantity of the target *CYP3A4* gene compared with the endogenous control (glyceraldehyde-3-phosphate dehydrogenase) was determined by the ΔΔCT method. The following primer sets were used in this study: CYP3A4 (5′-AGAAAGTCGCCTCGAAGATACA-3′ and 5′-GCTGGACATCAGGGTGAGTG-3′).

### 2.8. Pharmacokinetics and Statistical Analysis

The plasma concentration-time data of analytes were analyzed by compartment-independent approaches. The maximum plasma drug concentration (*C*_max⁡_) and time to *C*_max⁡_ (*T*_max⁡_) were directly obtained from the plasma concentration-time data. The elimination half-life (*t*_1/2_) was calculated as 0.693/*K*_*e*_, where *K*_*e*_, the elimination rate constant, was calculated via semilog regression on the terminal phase of the plasma concentration-time curve. The AUC from time 0 to infinity (AUC_0–*∞*_) was estimated as AUC_0–*t*_ + C_t_/*K*_*e*_, where C_t_ is the plasma concentration of the last measurable sample, and AUC_0–*t*_ was calculated according to the linear trapezoidal rule. Total plasma clearance (CL/F) was calculated as dose/AUC_0–*∞*_. The AUC metabolic ratio was calculated by dividing the AUC_0–*t*_ of 1-hydroxymidazolam by the AUC_0–*t*_ of midazolam. 

IC_50_ values (the concentration of inhibitor causing a 50% inhibition in enzyme activity) were calculated using Graphpad Prism 5.04 (GraphPad Prism, Inc. San Diego, CA, USA). For comparison of several groups against one control group, a one-way analysis of variance followed by Dunnett's test was performed. A *P* value < 0.05 was considered to be significant.

## 3. Results

All volunteers completed the study of three periods. 12 healthy male Chinese subjects with a mean age of 28 years (range, 26–38 years), a mean weight of 66.4 kg (range, 60–73.5 kg), and a mean height of 173 cm (range, 168–183 cm) participated in this study. 

### 3.1. Effect of CYP3A Activities by Danshen Ethanol Extract in Healthy Volunteers

**Figure 2:**
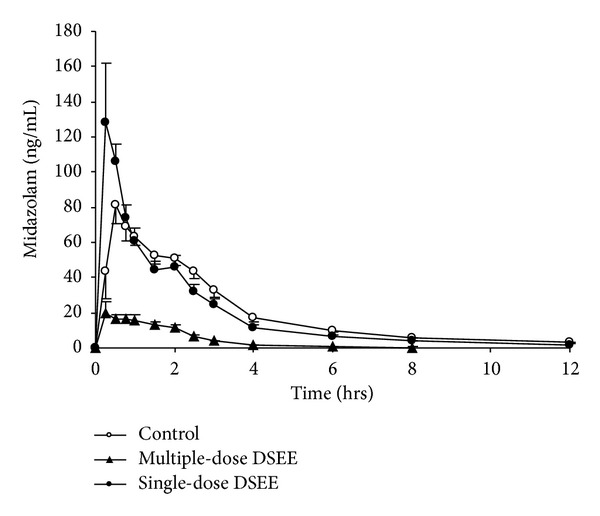
Mean (±SE, *n* = 12) plasma concentration of midazolam after the administration of a single dose of 15 mg of midazolam in 12 healthy volunteers before and after single- and multiple-dose co-administration of danshen ethanol extract (DSEE).

**Figure 3:**
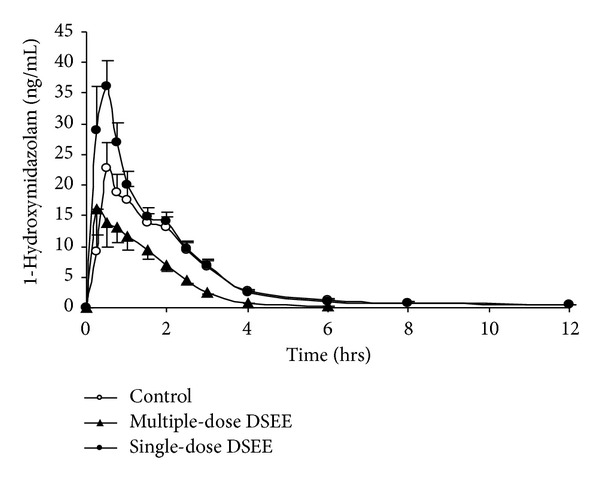
Mean (±SE, *n* = 12) plasma concentration of 1-hydroxymidazolam after the administration of a single dose of 15 mg of midazolam in 12 healthy volunteers before and after single- and multiple-dose coadministration of danshen ethanol extract (DSEE).

The mean plasma concentration-time profiles of midazolam and 1′-hydroxymidazolam before and after single or multidose administration of the danshen extract are presented in Figures 2 and 3.  Table 1 summarizes the pharmacokinetic parameters of midazolam, 1′-hydroxymidazolam and metabolic ratio of midazolam before and after single- or multi-dose of the danshen extract treatment. 

Regarding treatment with a single dose of the danshen extract, the *C*_max⁡_ of midazolam and 1′-hydroxymidazolam was increased by 80.7% (163.57 ± 86.36 ng/mL versus 95.17 ± 39.01 ng/mL) and 68.2% (45.04 ± 15.09 ng/mL versus 26.78 ± 11.08 ng/mL), compared with the control, respectively. 

After 10 days of the danshen extract intake, AUC_0–12_ of midazolam was decreased by 79.9% (42.24 ± 15.74 ng*·*h/mL versus 219.86 ± 64.67 ng*·*h/mL) compared with the control, and the clearance of midazolam was increased by 501.59% (393.71 ± 157.14 L/h versus 67.64 ± 20.05 L/h). After 10 days of treatment, the *C*_max⁡_ of midazolam was decreased by 66.6% compared with the control; *t*_1/2_ of midazolam was decreased by 43.8% (2.20 ± 0.90 h versus 4.20 ± 0.76 h). After 10 days of treatment, AUC_0–12_ of 1′-hydroxymidazolam was decreased by 44.50% (30.88 ± 15.09 ng*·*h/mL versus 56.21 ± 22.75 ng*·*h/mL) compared with the control, and *t*_1/2_ of 1′-hydroxymidazolam was decreased by 47.97% (1.72 ± 0.40 h versus 3.30 ± 0.62 h). However, the *C*_max⁡_ of of 1′-hydroxymidazolam was not significantly affected by 10-day treatment of the danshen extract.

After 10-day treatment with the danshen extract, AUC metabolic ratio of midazolam was increased by 35.64% compared with the control (0.77 ± 0.53 versus 0.26 ± 0.15).

### 3.2. Concentrations of Tanshinones in Human Plasma

After administration of a single dose (1 g) and multidose (1 g, three times each day) of the danshen extract, the pharmacokinetic parameters of tanshinones were listed in Table 2. 

### 3.3. Inhibition of CYP3A Activities by Danshen Ethanol Extract in Human Liver Microsomes

**Figure 4:**
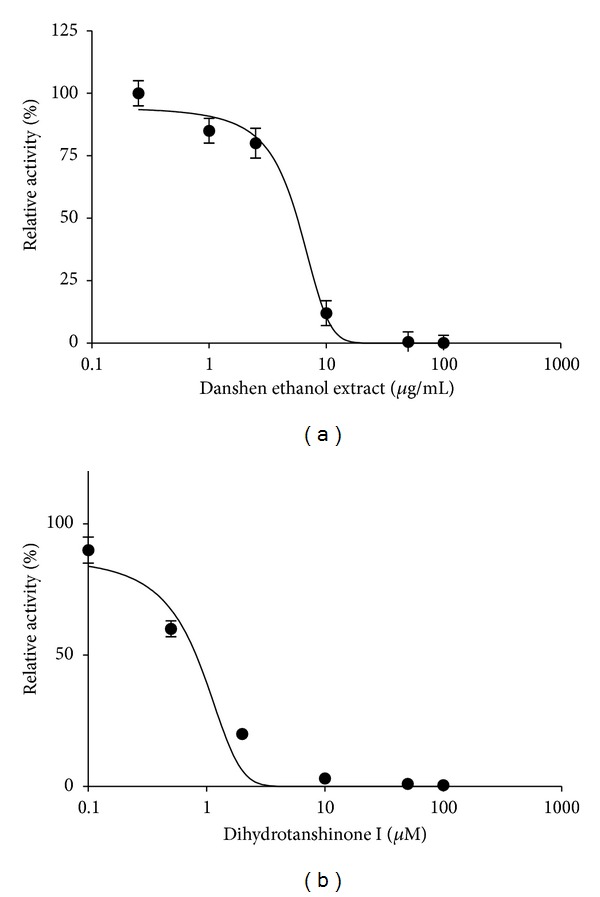
Inhibition of CYP3A4 activities by dihydrotanshinone I and danshen ethanol extract in human liver microsomes. Reactions were performed in the presence of midazolam (3.5 *μ*M) at various concentrations of danshen ethanol extract (0.2–100.0 *μ*g/mL) (a) and dihydrotanshinone I (0.5–100.0 *μ*M) (b), in the microsomes (0.2 mg/mL) and NADPH in a 100 mM phosphate buffer, pH 7.4 in a final volume of 200 *μ*L at 37°C for 5 min. Each point represents the mean of three separate experiments performed in triplicate, and the bar represents S.D.

To investigate whether the ethanol extract of danshen and which component(s) of the extract affected the catalytic activity of CYP3A, midazolam 1′-hydroxy reaction assays were conducted with various concentrations of ethanol extract of danshen and dihydrotanshinone I. The results showed that ethanol extract of danshen and dihydrotanshinone I had inhibition against CYP3A activities in HLMs with IC_50_ values of 8.6 *μ*g/mL and 1.2 *μ*M (Figure 4). 

### 3.4. Induction of CYP3A4 mRNA by Danshen Components in Human Hepatocytes

**Figure 5:**
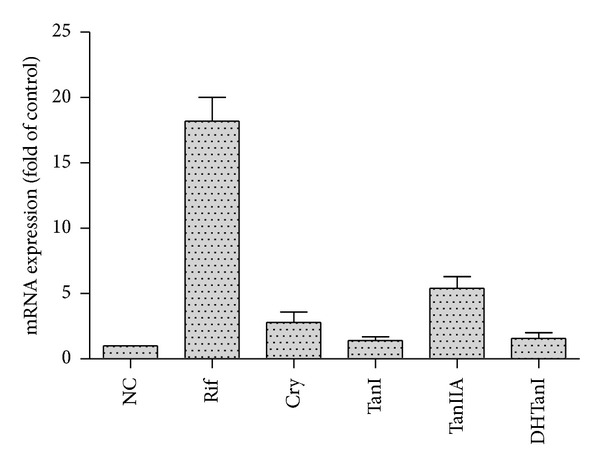
Induction of CYP3A4 mRNA by tanshinone I (TanI), cryptotanshinone (Cry), tanshinone IIA (Tan IIA), and dihydrotanshinone I (DHTanI). Human hepatocytes were exposed to tanshinone I (2 *μ*M), cryptotanshinone (2 *μ*M), tanshinone IIA (2 *μ*M), dihydrotanshinone I (2 *μ*M), or 25 *μ*M rifampin (PC) for 3 days. CYP3A4 mRNA levels were measured with reverse transcription real-time PCR. These data were obtained from two independent experiments, and each experiment was performed in triplicate. Each column with bar represents the mean and S.D. The mean is expressed as fold induction over vehicle control (NC).

Hepatocytes were treated with DMSO (0.1%), tanshinones (2 *μ*M), and rifampicin (25 *μ*M) for 72 hours. After treatment, expressions of *CYP3A4* mRNA were determined. Levels of *CYP3A4* transcripts were induced 18.2-fold by rifampicin (25 *μ*M). At 2 *μ*M, levels of *CYP3A4* transcripts by tanshinone I, cryptotanshinone, tanshinone IIA, and dihydrotanshinone I increased by 1.4-, 2.8-, 5.2-, and 1.5-fold, respectively (Figure 5).

## 4. Discussion

The ethanol extract of danshen in capsule dosage form represents a class of danshen product rich in lipophilic constituents in clinical practice. To our knowledge, this is the first report to evaluate the effect of the ethanol extract of danshen on the in vivo CYP3A activity in healthy volunteers. Midazolam (MDZ) is a widely accepted probe drug for CYP3A phenotype [15]. MDZ is rapidly metabolized by CYP3A to 1′-hydroxymidazolam (1′-OHMDZ) and, to a smaller extent, to 4-hydroxymidazolam (4-OHMDZ) and 1,4-dihydroxymidazolam (1,4-OHMDZ), and it is further metabolized to glucuronide conjugates by UDP-glucuronyltransferase (UGT) [16]. 

In this study, an 80.7% increase in the *C*_max⁡_ of midazolam occurred with single-dose administration of the ethanol extract of danshen. This increase was not reflected in the AUC_0–12_, CL/F, or *t*_1/2_. Although midazolam is classified into BCS class I, with high membrane permeability [17], its oral bioavailability is only 24 to 46% in humans [18]. Gorski found that the oral bioavailability of midazolam was almost entirely determined by CYP3A activity in the small intestine [19]. So, the increase in *C*_max⁡_ could attribute to inhibition of CYP3A enzymes in the small intestine. 

The danshen extract contains cryptotanshinone and dihydrotanshinone I, and the content of dihydrotanshinone I was 5 times lower than cryptotanshinone in the preparation. We reported that cryptotanshinone could activate midazolam 1-hydroxylation in human liver microsomes [20]. After administration of 1 g danshen extract, the concentration of cryptotanshinone in the liver and intestine can reach the concentration of 2 *μ*M to activate midazolam 1-hydroxylation. In contrast, dihydrotanshinone I absorbed into liver cannot reach the concentration with inhibition against CYP3A4. Therefore, the inhibition of CYP3A4 in intestine can be offset by cryptotanshinone in liver activation effect. It can be one of reasons why the AUC of midazolam was not obviously changed after single-dose treatment of the danshen extract. An in vitro study found that the content of the ethanol extract of danshen had a significant inhibitory effect on CYP3A-mediated 1-hydroxymidazolam metabolism with IC_50_ 8.6 *μ*g/mL in human liver microsomes. Although tanshinone IIA, tanshinone I, and cryptotanshinone in the danshen extract had no significant inhibitory effect individually [14, 20], dihydrotanshinone I (the danshen extract containing 13 mg dihydrotanshinone per 1 g) was a strong inhibitor of CYP3A4 with IC_50_ 1.20 *μ*M. The results were consistent with Wang's report [9]. After treatment with 1 g of the danshen extract, there were higher concentrations of dihydrotanshinone I in the small intestine beyond inhibition concentration (IC_50_) toward intestinal CYP3A. 

By contrast, after 10-day intake of the danshen extract, the clearance was increased, and the *C*_max⁡_,  AUC_0–*∞*_, and *t*_1/2_ were decreased compared with baseline. It suggests that both presystemic processes and systemic elimination of midazolam are altered by prolonged intake of the ethanol extract of danshen. And it indicates that intestinal and hepatic CYP3A are induced by multi-dose of the extract administration. After 10 days of treatment, *t*_1/2_ of 1-OHMDZ was also reduced by 54.1% compared with control, suggesting that UGTs could be induced to enhance the glucuronidation of 1-OHMDZ by the danshen extract. This is also evidenced by metabolic ratio of midazolam. 

It was the first report to study induction of tanshinones on CYP3A4 using primary human hepatocytes. A cutoff value of 4-fold increase in mRNA levels for induction compared with those found in negative control was applied for assessing CYP3A4 mRNA expression [21]. The first in vitro finding in primary human hepatocytes demonstrates that tanshinone IIA can induce the expression of the CYP3A4 gene which was increased up to the cutoff value. Cryptotanshinone exhibits small increase (2.8-fold) of *CYP3A4* mRNA which does not reach the cutoff value. The cutoff value may vary among different laboratories because of the variability among hepatocyte lots. However, tanshinone I and dihydrotanshinone I at 2 *μ*M do not cause significant increase of CYP3A4 mRNA. The results were consistent with Yu's findings in the reporter gene [8]. After pretreatment with higher dose of the danshen extract rich in tanshinone IIA and cryptotanshinone for 10 days, there were much higher tanshinones concentrations in the volunteers' guts to induce intestinal CYP3A, and there were also effective enough tanshinones concentrations in the liver which can induce the hepatic CYP3A even with less than 10 ng/mL of plasma concentration. This finding supports the hypothesis that relevant inhibition can only be achieved locally in the gut, whereas the concentration in the liver is sufficient for PXR activation and subsequent induction of metabolism. It is clear that dihydrotanshinone I can inhibit CYP3A, while other constituents such as tanshinone IIA and cryptotanshinone can mediate the inductive response in danshinone extract.

We reported that administration of danshen tablets for 2 weeks (4 tablets each time, 3 times a day) in healthy volunteers according to one-sequence crossover design caused 35.4% increase in apparent oral clearance. In this study, the clearance was increased by 501.5%. The extent CYP3A induction in intestine by the danshen extract rich in tanshinones in this study was 14.2 times higher than that of the danshen extract with low tanshinones content which contains cryptotanshinone 1.2 mg and tanshinone IIA 1.6 mg in each dose [22]. The contents of the three tanshinones in danshen tablet were 50 times lower than the danshen capsule. So, the extent of the induction of danshen extract toward CYP3A was shown to be dependent on dose of tanshinones. 

In addition to CYP3A, the nuclear receptors of UGT are also targets of PXR [23–26]. So, tanshinone IIA and cryptotanshinone could activate PXR and consequently induce the expression of the *UGT* gene. Since more than 40% of clinically used drugs are catalyzed by CYP3A and with further biotransformation by UGT, the two drug metabolising enzymes can be induced by the danshen extract to promote their substrates conversion to more polar derivatives which can be readily excreted. PXR has also been shown to play key roles in the regulation of several other inducible *CYP2C9, CYP2C19, P-gp, MRP2*, *sulfate transferase (ST),* and other drug metabolisming enzymes and transporter genes [23–26]. So, the inductive effect of tanshinones on these metabolic enzymes also should be researched. 

Drugs that are substrates for CYP3A-mediated metabolism are likely to be potential candidates for drug-herb interactions [27]. The duration and dosage of exposure to the ethanol extract of danshen appear to be critical for drug-danshen interactions. An increase in the plasma drug concentration is possible during concomitant administration of the ethanol extract of danshen and prescribed drugs. By contrast, prolonged intake of the danshen extract followed by drug administration may result in subtherapeutic drug concentrations. Although we have shown that the ethanol extract of danshen has the potential to inhibit CYP3A4, particularly after single exposure at high concentrations, the inductive effect predominates with chronic exposure. It is suggested that caution should be taken when coadministrating the danshen extract rich in tanshinones with therapeutic drugs which are substrates for CYP3A4. 

## Figures and Tables

**Table 1:** Pharmacokinetic parameters of midazolam and 1-hydroxymidazolam after the administration of a single dose of 15 mg of midazolam in 12 healthy volunteers before and after single- and multiple-dose coadministration of the ethanol extract of danshen.

PK Parameter	Control	Single dose	*P* value*	Multiple dose	*P* value*
Midazolam					
*C*_max⁡_ (ng/mL)	95.17 ± 39.01	163.57 ± 86.36	0.00	39.55 ± 18.52	0.00
*T*_max⁡_ (h)	0.69 ± 0.60	0.35 ± 0.13	0.07	0.79 ± 0.65	0.68
AUC_0–12_ (ng·h/mL)	219.86 ± 64.67	213.85 ± 86.31	0.70	42.24 ± 15.74	0.00
AUC_0–*∞*_ (ng·h/mL)	221.76 ± 63.78	218.15 ± 83.22	0.65	44.55 ± 17.68	0.00
*t*_1/2_ (h)	4.20 ± 0.76	4.01 ± 1.08	0.61	2.20 ± 0.90	0.00
CL/F (L/h)	67.64 ± 20.05	75.78 ± 27.94	0.18	393.71 ± 157.14	0.00
1-Hydroxymidazolam					
*C*_max⁡_ (ng/mL)	26.78 ± 11.00	45.04 ± 15.09	0.01	20.48 ± 13.70	0.18
*T*_max⁡_ (h)	0.57 ± 0.20	0.61 ± 0.39	0.12	0.46 ± 0.13	0.74
AUC_0–12_(ng·h/mL)	56.21 ± 22.75	66.62 ± 25.24	0.12	30.88 ± 15.09	0.00
*t*_1/2_ (h)	3.30 ± 0.62	3.29 ± 0.78	0.16	1.72 ± 0.70	0.00
AUC_(1OHmdz)_/AUC_(mdz)_	0.26 ± 0.15	0.32 ± 0.15	0.08	0.77 ± 0.53	0.00

Data are presented as mean ± SD. *P values are given for the differences with respect to control. The data were analyzed using a one-way analysis of variance with post hoc Dunnett's procedure.

**Table 2:** Pharmacokinetic parameters of tanshinons in 12 healthy volunteers after single- and multiple-dose administration of the ethanol extract of danshen.

PK parameter	Tanshinone I	Cryptotanshinone	Tanshinone IIA	Dihydrotanshinone I
*C*_max⁡_ (ng/mL)	11.52 ± 9.90	3.44 ± 1.32	4.00 ± 2.79	0.85 ± 0.59
*C*_trough_ (ng/mL)	3.09 ± 1.09	0.76 ± 0.23	1.36 ± 0.52	0.20 ± 0.15
*C*_ssmax_ (ng/mL)	18.10 ± 16.44	3.33 ± 1.85	3.98 ± 1.97	1.00 ± 0.56

Data are mean value ± SD.

*C*_max⁡_: peak plasma concentration after single-dose administration of the danshen extract; *C*_trough_: plasma trough concentration at the end of a dosing interval after multiple-dose administration of the danshen extract; *C*_ssmax_: peak plasma concentration at the end of a dosing interval after multiple-dose administration of the danshen extract.
